# Changes in haptoglobin genotype-based gene expressions upon the observance of dawn-to-dusk intermittent fasting: a prospective cohort study on overweight and obese individuals

**DOI:** 10.3389/fnut.2024.1409344

**Published:** 2024-10-01

**Authors:** Mohamed I. Madkour, Rasha E. Hassan, Naglaa M. Sherif, Samir Awadallah, Nada M. Farahat, Dana N. Abdelrahim, Fatima A. AlHasan, Jalal Taneera, MoezAlIslam E. Faris

**Affiliations:** ^1^Department of Medical Laboratory Sciences, College of Health Sciences, University of Sharjah, Sharjah, United Arab Emirates; ^2^Research Institute of Medical and Health Sciences (RIMHS), Center of Excellence for Public Health, University of Sharjah, Sharjah, United Arab Emirates; ^3^Biochemistry Department, Faculty of Science, Ain Shams University, Cairo, Egypt; ^4^Department of Medical Laboratories, College of Allied Medical Sciences, Zarqa University, Zarqa, Jordan; ^5^Department of Medical Laboratory Medicine, King Fahd Hospital, College of Applied Medical Sciences, Imam Abdulrahman Bin Faisal University, Dammam, Saudi Arabia; ^6^College of Medicine, Center of Excellence for Precision Medicine, Research Institute of Medical and Health Sciences (RIMHS), University of Sharjah, Sharjah, United Arab Emirates; ^7^Department of Clinical Nutrition and Dietetics, Faculty of Allied Medical Sciences, Applied Science Private University, Amman, Jordan

**Keywords:** calorie restriction, time-restricted eating, oxidative stress, inflammation, nutritional genomics, Gene polymorphisim

## Abstract

**Introduction:**

Intermittent fasting (IF) has been reported to be involved in ameliorating oxidative stress and lessening the systemic-low grade inflammation that predisposes to chronic diseases. Gene polymorphism is currently a main determining factor for the metabolic responses to different dietary and lifestyle modifications.

**Methods:**

The current study was designed to explore the effect of observing four-week, dawn to dusk IF by participants with obesity on gene expression of the anti-inflammatory *CD163*, oxidative stress, and bioenergetics enzymes (*SOD2*, *Nrf2*, and *TFAM*), as well as metabolic and cellular regulatory genes (*SIRT1* and *SIRT3*). Further, the study aimed to find out how haptoglobin (Hp) polymorphism modulates gene expression of the aforementioned genes and to determine changes in relative gene expressions of the aforementioned six genes based on Hp polymorphism in response to IF. Haptoglobin genotype was determined for the study subjects, and gene expressions were determined using qPCR. Gene expressions were assessed before and at the end of four consecutive weeks, dawn to sunset IF.

**Results:**

The expressions of *CD163*, *SOD*, *NfF2*, and *TFAM* genes have significantly increased at the end of IF. At the same time, *SIRT3* significantly decreased, implying that observing four consecutive weeks of dawn-to-dusk IF may enhance antioxidative stress response and reduce systemic inflammation.

**Conclusion:**

Participants with genotypes Hp2-1 and Hp2-2 revealed upregulation of the antioxidant genes in response to the metabolic stress induced by IF compared with Hp1-1, implying that Hp polymorphism plays a key role in shaping the body’s response to dietary modifications such as fasting.

## Introduction

Obesity is a significant global health burden that adversely affects human health due to its numerous disease complications (1). Many of these complications are driven by obesity-induced oxidative stress (OS) and low-grade systemic inflammation, including insulin resistance, diabetes, infertility, asthma, and liver disease (2).

Intermittent fasting (IF) has grasped significant attention in recent years as a potential strategy for weight management and metabolic health improvement. However, numerous studies have explored the effects of IF on metabolic parameters, which include reducing the risk of obesity-related cardiometabolic diseases, lowering oxidative stress, improving lipid profiles, inhibiting atherosclerosis, and mitigating inflammation (3, 4). However, limited research has investigated the genetic and molecular mechanisms underlying these effects, particularly in the context of specific genetic variations.

Among the various forms of IF, Ramadan intermittent fasting (RIF) involves abstaining from food and drink from dawn to sunset. Observed by about 1.5 billion Muslims out of more than two billion, RIF mandates complete abstention during this period (5, 6). This unique form of fasting has provided researchers with a natural model to study the physiological and molecular impacts of IF on human health. Numerous studies have examined the effects of observing RIF on different aspects related to obesity and metabolism, including body weight (7) and body fatness (8), with particular attention toward visceral adiposity (9), OS and inflammation markers (10–12), glucometabolic regulation (13), liver disease, metabolic syndrome and cardiometabolic risk factors (7, 14), circadian rhythm and eating regulation hormones (15), and sleep quality (16).

Haptoglobin (Hp) is a plasma protein primarily known for its role in binding free hemoglobin (Hb) and preventing oxidative damage. The Hp gene exhibits genetic polymorphism, resulting in three primary genotypes: Hp1-1, Hp2-1, and Hp2-2 (17). These genotypes differ in their biochemical properties and functional capacities, which may influence individual responses to metabolic stress and oxidative challenges (17). The variation in the Hp genotype has been implicated in various health conditions, including cardiovascular diseases, diabetes, and inflammatory disorders (18–20). Compared to Hp2-1 and Hp2-2, which have a lower Hp-Hb complex stability, the Hp2-2 genotype is associated with an increased OS and proinflammatory response, making it a risk factor for inflammation (18, 19, 21). People with different Hp genotypes have been shown to respond differently to dietary modifications such as IF, where Hp2-2 individuals had a higher anti-inflammatory response than Hp2-1 and Hp1-1 individuals upon the observance of RIF (22). Thoroughly in our previous study, we reported that 4 weeks of dawn-to-dusk-RIF was associated with significantly altered anthropometric, metabolic, and inflammatory markers, with variable responses observed based on Hp polymorphisms. According to our findings, RIF is associated with reduced low-grade systemic inflammation, enhanced anti-inflammatory markers, and ameliorated obesity-related health conditions (22).

With the rising interest in the effects of IF on weight regulation and overall health and the integration between dietary modifications and gene expressions as part of the study of nutritional genomics, it is crucial to examine how individuals with different Hp genotypes respond to dietary modifications like IF. Additionally, it is important to explore how IF influences the expression of genes responsible for inflammation, OS, and cellular regulation and metabolism. Therefore, the primary objective of this study is to examine how gene expressions change upon the observance of a four-week, dawn-to-sunset RIF among overweight/obese participants with different Hp genotypes. Specifically, the study investigates how Hp modulates the expression of genes related to inflammation and OS, such as *CD163*, *SOD2*, *Nrf2*, and *TFAM*, and metabolic and cellular regulatory genes like *SIRT1* and *SIRT3*. We hypothesize that overweight/obese participants with different Hp genotypes will respond differently to RIF and that the six gene expressions (*CD163*, *SOD2*, *Nrf2*, *TFAM*, *SIRT1*, and *SIRT3*) will vary accordingly.

Our previous work examined general changes in the expression of five genes (*SOD2*, *Nrf2*, *TFAM*, *SIRT1*, and *SIRT3*) during RIF, regardless of Hp gene polymorphism (22). Hence, the novelty of this current study lies in analyzing the expression of these six genes based on Hp gene polymorphism, thereby demonstrating how gene expressions respond differently to dietary modifications like IF among overweight/obese individuals according to their Hp genetic profile.

In a large scope, the study focuses on examining the interplay between genetic predisposition and environmental factors, such as dietary patterns and fasting, in influencing metabolic health outcomes. The investigation of Hp genotype-based gene expressions during IF can provide insights into the molecular mechanisms driving the metabolic benefits of this dietary practice. By examining gene expression profiles across different Hp genotypes, this study aims to elucidate the genotype-specific molecular pathways that mediate the beneficial effects of IF. This research contributes to the development of our understanding of gene-nutrient interactions in the context of the nutritional genomics field of study.

## Methods

### Study design

A prospective cohort design was employed in this study to examine the variations in OS, cell regulatory, and metabolic gene expression resulting from Ramadan intermittent fasting (RIF) among participants with overweight/obesity and three distinct haptoglobin (Hp) genotypes. The study was conducted over two Ramadan fasting months in two consecutive years, specifically during May and June of 2017 and 2018. Data collection occurred at two different time points: baseline, which took place 2 to 7 days before the start of RIF, and at the end of the fourth week of the Ramadan month, following 28 to 30 consecutive days of dawn-to-sunset RIF. Throughout the fasting period, which lasted approximately 15 h per day, participants refrained from oral intake, including both food and water, from dawn until sunset. No specific dietary or physical activity guidelines were given to the participants at any stage of the study. In accordance with Islamic fasting laws, menstruating women were exempted from Ramadan fasting during their menstrual period, resulting in a shorter fasting period for female participants (23–25 days) compared to male participants (28–30 days).

### Participant selection

A convenience sampling method was applied in this study. Upon announcing the research through social media, institutional emails, and personal communications, individuals who expressed their interest in observing Ramadan fasting and visited the University Hospital Sharjah (UHS)/UAE for screening were recruited. The study adhered to the principles of the Declaration of Helsinki and received approval from the UHS Research Ethics Committee (Reference No. REC-16-05-11-01). Each participant was given an information sheet detailing the research plan, objectives, and requirements for involvement, and they provided signed informed consent before participating. To be eligible for the study, male or female participants had to be overweight/obese (body mass index, BMI >25 kg/m^2^), willing to fast during Ramadan, and willing to take part in the research. Data collection involved a self-report questionnaire covering the medical history and demographic details, which were gathered through face-to-face interviews conducted by trained research assistants. Exclusion criteria encompassed a history of metabolic syndrome, diabetes, or cardiovascular disease; neuro-psychiatric patients on regular medications; individuals following a weight-reducing diet; those who had undergone bariatric surgery within the last 9 months before starting RIF; and pregnant or premenopausal women.

As an observational, non-interventional, or experimental study, it is acceptable to use a self-control, pre-post design to demonstrate the effects of dietary or lifestyle modifications on specific health outcomes. In the case of the Ramadan fasting model, having a non-fasting control group from the same ethnic population during this short period of the Holy month of Ramadan is very challenging. Being a religious ritual that is strictly observed and spiritually significant for the Muslim community, it is very challenging to secure non-fasting individuals. Even non-religious people often adhere to fasting during Ramadan out of empathy and solidarity with the fasting community. This challenge is further emphasized in gene testing studies, where ethnic background plays a key role in determining gene expression levels (23).

### Blood samples collection

Blood samples were obtained from the participants after an 8–10 h fasting period at both time intervals. A total of 10 mL of blood was collected during each of the two-time intervals. To maintain consistency in the duration of fasting and eliminate the impact of timing and dietary intake on measured biochemical parameters, the blood samples were collected between 11 am and 1 pm on both occasions. After collection, the blood samples were split into two aliquots. One aliquot was subjected to centrifugation at 2,500 rpm for 15 min within 1 h of collection. The resulting serum was then divided into coded aliquots and stored at −80°C until it was ready for use in biochemical analyses. The second aliquot was reserved for RNA extraction.

### Haptoglobin genotype determination

Hp genotype distribution was determined using 8% vertical polyacrylamide gel electrophoresis (24). Briefly, the Hp-hemoglobin (Hp-Hb) complex solution was prepared by adding 5 mL of 10% Hb A to 40 mL of the sample buffer (50% glycerol), followed by 10 mL of the sample serum. Electrophoresis was then run at room temperature for 4 h at 130 volts. The gel was removed from the apparatus and immersed in benzidine solution for 30 min to visualize the Hp bands. The benzidine solution was prepared by dissolving 0.2 g of benzidine in 250 mL boiling water. Just before staining, 1.5 mL glacial acetic acid and 0.6 mL H_2_O_2_ were added to the benzidine solution (25).

#### Quantitative real-time PCR

First, blood RNA extraction was performed using the Norgen Biotek Total RNA Purification kit as per manufacturer instructions. The total RNA yield was assessed spectrophotometrically using (Nanodrop 2000, Thermo Scientific) by measuring the A260/A280 ratio to check the purity, where only the samples with a 1.6 ratio and above were chosen. Norgen’s TruScript^™^ First Strand cDNA Synthesis Kit has been used for our sample to get a cDNA complementary strand, as per the manufacturer’s instructions. qPCR reaction was performed at the volume of 20 μL, including 100 ng of cDNA with QuantiTect SYBR Green PCR mixture (Qiagen, Germany). The cycling conditions included initial activation of the polymerase for 15 min at 95°C, followed by 45 cycles of 15 s denaturation at 94°C, annealed at 55°C for 30 s, followed by extension at 72°C for 30 s. The primers used are listed in Table 1. For each sample, the expression of each gene was normalized to the housekeeping (GAPDH) at the same time point. The data were compared with a pool of six healthy controls with normal BMI (18.5–24.9 kg/m^2^) at each time point. The relative expression was shown as fold change according to Livak and Schmittgen (26) and presented as mean and standard deviation (SD), as described elsewhere (27).

**Table 1:** Forward and reverse primers for the main six genes tested in the study.

Gene	Primer	References
*CD163*	Forward: 5′-CCAGTCCCAAACACTGTCCT-3′	(46)
	Reverse: 5′-ATGCCAGTGAGCTTCCCGTTCAGC-3′	
*Nrf2*	Forward: 5′-ATGGATTTGATTGACATACTTT	(47)
	Reverse: 5′-ACTGAGCCTGATTAGTAGCAAT	
*SOD2*	Forward: 5′-GCTCCGGTTTTGGGGTATCTG	(48)
	Reverse: 5′-GCGTTGATGTGAGGTTCCAG	
*TFAM*	Forward: 5′-ATGGCGTTTCTCCGAAGCAT	(49)
	Reverse: 5′-CAGATGAAAACCACCTCGGTAA	
*SIRT1*	Forward: 5′GCCTCACATGCAAGCTCTAGTGAC	(50)
	Reverse: 5′-TTCGAGGATCTGTGCCAATCATAA	
*SIRT3*	Forward: 5′-ACCCAGTGGCATTCCAGAC	(51)
	Reverse: 5′-GGCTTGGGGTTGTGAAAGAAG	
*GAPDH*	Forward: 5′-CCAGGTGGTCTCCTCTGACTTC	(52)
	Reverse: 5′-TCATACCAGGAAATGAGCTTGACA	

### Statistical analysis

Statistical analyses were performed using SPSS 29 (IBM, Armonk, NY, United States). The mean and standard deviation (SD) were calculated for continuous variables and the percentage (%) for categorical variables. The normality distribution of the data was tested using Kolmogorov Smirnov. The change was calculated as [the endpoint value − baseline value], and the % change was calculated as [(endpoint value − baseline value) /baseline value] × 100%. The *p*-value for the trend was analyzed using a linear trend test. The Wilcoxon signed-rank test for paired samples was used to compare changes within groups over the time course of the study. Changes in variables within groups are presented as adjusted *p*-values derived from a general linear model after adjusting for the baseline age, sex, waist circumference, and total caloric intake. The significance was considered at *p*-values <0.05.

## Results

### Basic demographic, anthropometric, and inflammatory characteristics

A total of 114 overweight and obese participants (75 males and 39 females, 38.7 ± 11.7 years) with a mean BMI of 30.4 ± 5.09 kg/m^2^ were recruited in this observational study. Changes in anthropometric, metabolic, and inflammatory markers before and at the end of RIF are shown in our previously published work of Madkour et al. (22). According to the data in that work, the patients experienced a significant average weight loss of 1.6% of their baseline body weight. Additionally, there were significant decreases in other anthropometric measurements, including body mass index, body fat percent, fat mass, fat-free mass, muscle mass, visceral fat surface area, waist circumference, hip circumference, and waist: hip ratio at the end of the fasting period compared to their levels before fasting. Furthermore, there were notable decreases in serum triglycerides, total cholesterol, and LDL levels. At the same time, there was a significant increase in serum HDL levels at the conclusion of the fasting month for the entire population. At the end of the month of fasting, the study individuals showed a notable decrease in the levels of serum Hp, IL-6, and TNF-α compared to their levels before fasting. At the end of Ramadan, the study participants exhibited a considerable increase in serum CD163 and IL-10 levels. These results also revealed that Hp2-2 was the most frequently abundant genotype among the study participants, representing 48.24% (*n* = 55), followed by Hp 2-1 (46.5%, *n* = 53), and by Hp 1-1 (5.26%, *n* = 6).

### Relative gene expression according to the haptoglobin polymorphism

**Figure 1:**
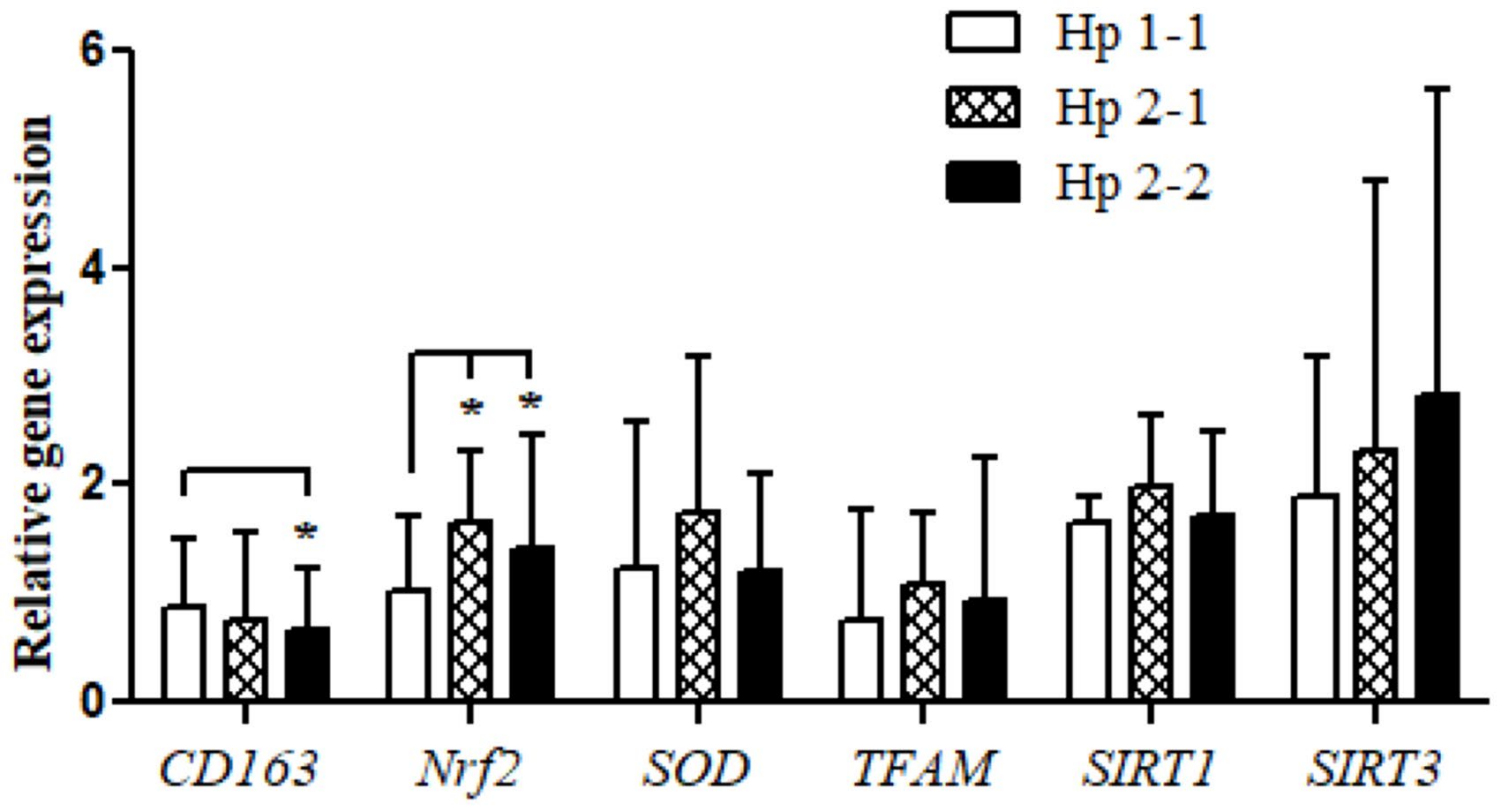
Relative gene expressions for the *CD163*, antioxidant (*Nrf2*, *SOD2*, and *TFAM*), and metabolic and cellular regulatory (*SIRT1* and *SIRT3*) genes according to the Hp genotype for the study subjects. ^*^p < 0.05.

Relative gene expression of CD163, antioxidant enzymes (*Nrf2*, *SOD2*, and *TFAM*), and metabolic and cellular regulatory (*SIRT1* and *SIRT3*) genes in overweight and obese participants in comparison to counterpart gene expressions for controls based on their Hp genotype distribution as depicted in Figure 1. The gene expression of CD163 represents a trend of value decrements from Hp1-1 to Hp2-1 to Hp2-2 with a significant difference between Hp2-2 compared to Hp1-1. Also, at the same pattern, the results show a higher significance of *Nrf2* gene expression in Hp2-1 and Hp2-2 in comparison to Hp1-1 individuals with overweight/obesity. In contrast, no significant differences were observed in gene expressions between Hp1-1, Hp2-1, and Hp2-2 in the antioxidant enzymes (*SOD2* and *TFAM*) and metabolic and cellular regulatory genes (*SIRT1* and *SIRT3*).

### Changes in relative gene expressions before and after IF

**Figure 2:**
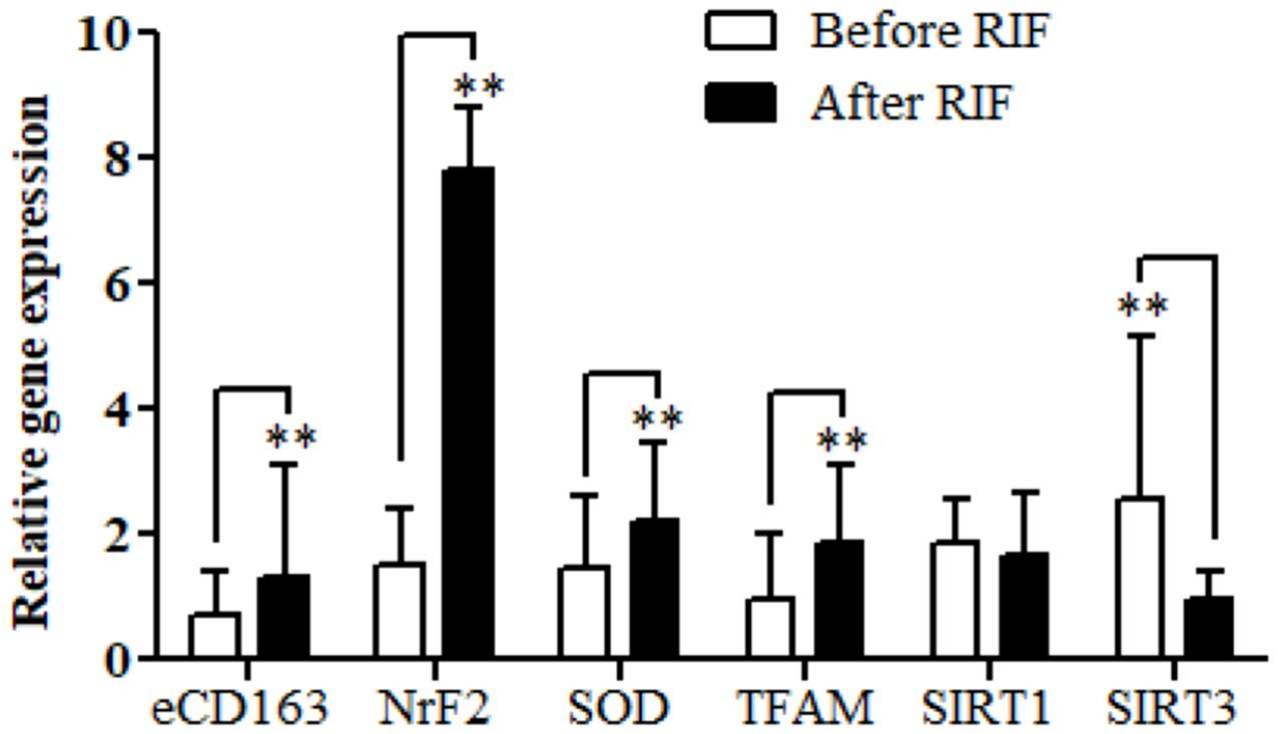
Changes in relative gene expressions for *CD163*, antioxidant (*SOD2*, *Nrf2*, and *TFAM*), and metabolic and cellular regulatory (*SIRT1* and *SIRT3*) genes before and after observing RIF month. ^**^p < 0.001.

Relative gene expressions in participants with overweight and obese for *CD163*, antioxidant (*SOD2*, *Nrf2*, and *TFAM*), and metabolic and cellular regulatory (*SIRT1* and *SIRT3*) genes assessed for study participants are shown in Figure 2. The results of the expressions showed a highly significant increase in the *CD163*, *Nrf2*, *SOD2*, and *TFAM* genes in the study participants at the end of Ramadan compared with the pre-fasting levels. In contrast, the relative gene expression of the metabolic and cellular regulatory *SIRT3* gene showed a significant reduction in the study participants at the end of RIF in comparison with the levels before RIF. Moreover, *SIRT1* gene expression represented a clear decremental trend at the end of RIF in comparison to the expressions before RIF, but without a statistically significant value (Figure 2).

### Changes in relative gene expression before and after RIF according to Hp genotype

**Figure 3:**
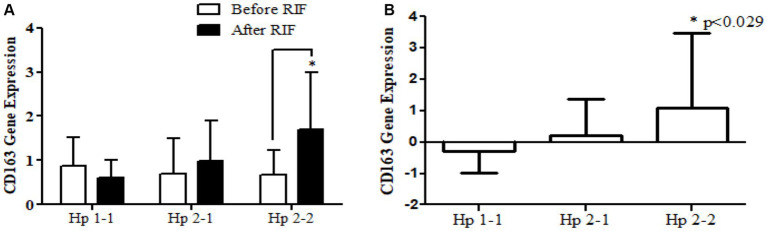
**(A)** Changes in CD163 gene expression before and after RIF based on Hp genotype. **(B)** Mean of differences in CD163 gene expressions before and after RIF based on Hp genotype. ^*^p < 0.05.

**Figure 4:**
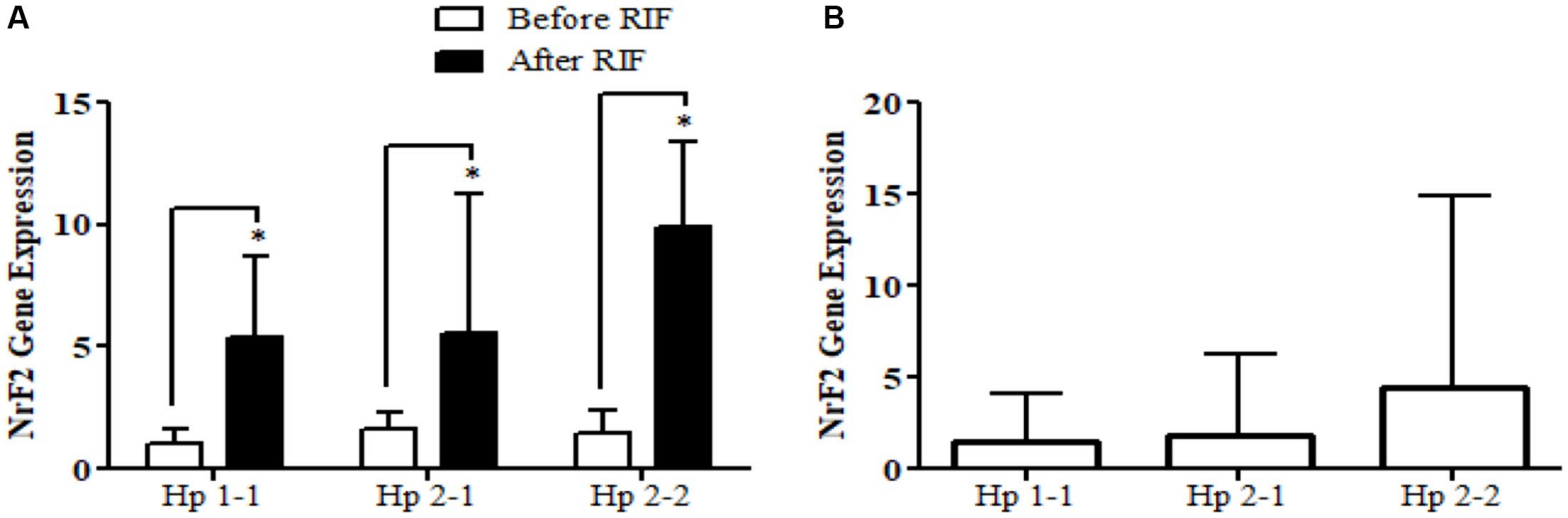
**(A)** Changes in *Nrf2* gene expression before and after RIF based on Hp genotype. **(B)** Mean of differences in *Nrf2* gene expressions before and after RIF based on Hp genotype.

Relative gene expression in obese participants of *CD163*, antioxidant (*SOD2*, *Nrf2*, and *TFAM*), and metabolic and cellular regulatory (*SIRT1* and *SIRT3*) genes in response to RIF-based Hp genotype are resented in Table 2. A highly significant increase in *CD163* gene expression for those participants with Hp2-2, with no such significant differences was observed in participants with Hp1-1 and Hp2-1 polymorphism genotypes at the end of Ramadan fasting in comparison with the pre-fasting expression levels (Figure 3). Besides, the mean difference in the expression of the *CD163* gene showed a significant increase between Hp polymorphism Hp2-2 in comparison with Hp1-1 groups. Likewise, *Nrf2* gene expression showed significant increases in participants with Hp1-1, Hp2-1, and Hp2-2 genotypes at the end of RIF in contrast with the pre-fasting levels (Figure 4). Also, the mean of difference for *Nrf2* gene expression between the Hp polymorphism genotype revealed a trend of increment associated with the expression through Hp1-1, Hp 2-1, and Hp 2-2, respectively, but without significant changes.

**Table 2:** Changes in relative gene expressions before and after RIF based on Hp genotype.

Gene	Hp1-1 (*n* = 6)		Hp2-1 (*n* = 53)		Hp2-2 (*n* = 55)	
	Before RIF	End of RIF	Before RIF	End of RIF	Before RIF	End of RIF
*CD163*	0.88 ± 0.66	0.60 ± 0.41	0.77 ± 0.80	0.99 ± 0.92	0.67 ± 0.57	1.75 ± 2.36*
*Nrf2*	1.04 ± 0.68	5.46 ± 3.31*	1.67 ± 0.68	5.52 ± 5.88*	1.44 ± 1.04	9.92 ± 13.56*
*SOD2*	1.23 ± 1.39	0.71 ± 0.01	1.74 ± 1.43	2.04 ± 1.68	1.21 ± 0.92	2.49 ± 2.24*
*TFAM*	0.76 ± 1.04	1.57 ± 1.55	1.07 ± 0.67	1.73 ± 1.05*	0.93 ± 1.33	2.01 ± 1.47*
*SIRT1*	1.66 ± 0.26	1.53 ± 0.16	1.99 ± 0.66	1.67 ± 0.90	1.73 ± 0.77	1.64 ± 1.25
*SIRT3*	1.91 ± 1.29	0.87 ± 0.56	2.32 ± 2.51	1.01 ± 0.44*	2.85 ± 2.80	0.97 ± 0.44*

*CD163*, Cluster of Differentiation-163 gene; *Nrf2*, nuclear factor Erythroid 2–related factor 2; *SOD2*, superoxide dismutase 2; *TFAM*, Mitochondrial transcription factor A; *SIRT1*, silent information regulator sirtuin 1; *SIRT3*, NAD-dependent deacetylase sirtuin-3. *Indicates significance.

**Figure 5:**
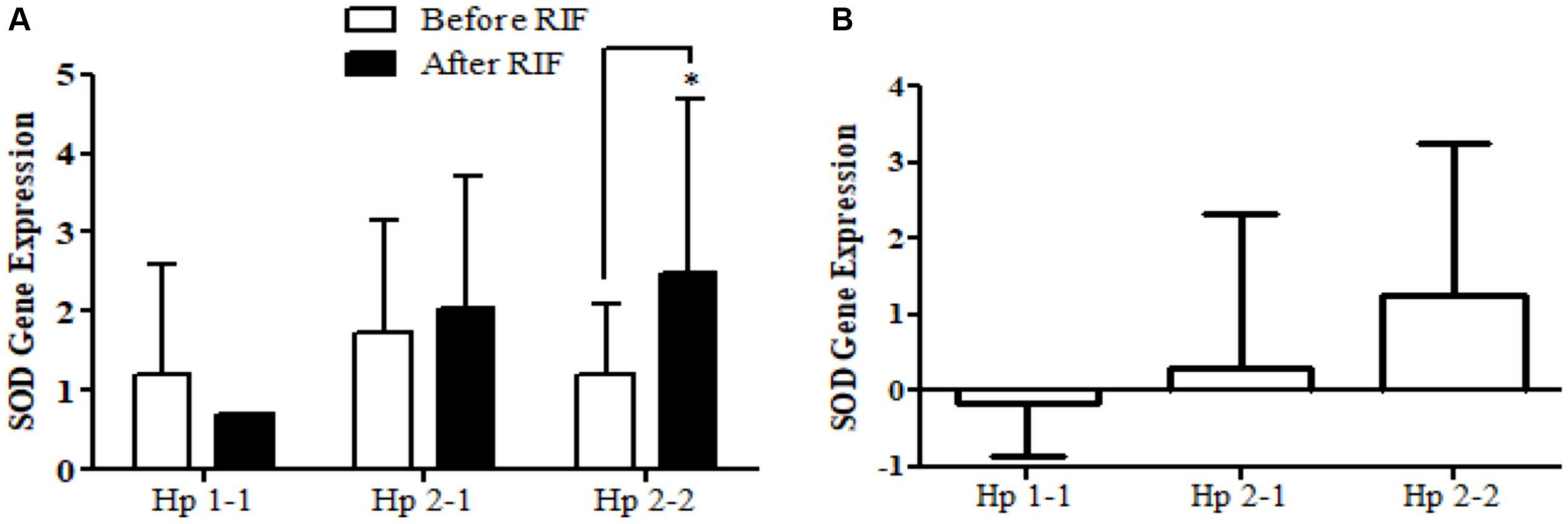
**(A)** Changes in *SOD2* gene expression before and after RIF based on Hp genotype. **(B)** Mean of differences in *SOD2* gene expressions before and after RIF based on Hp genotype.

**Figure 6:**
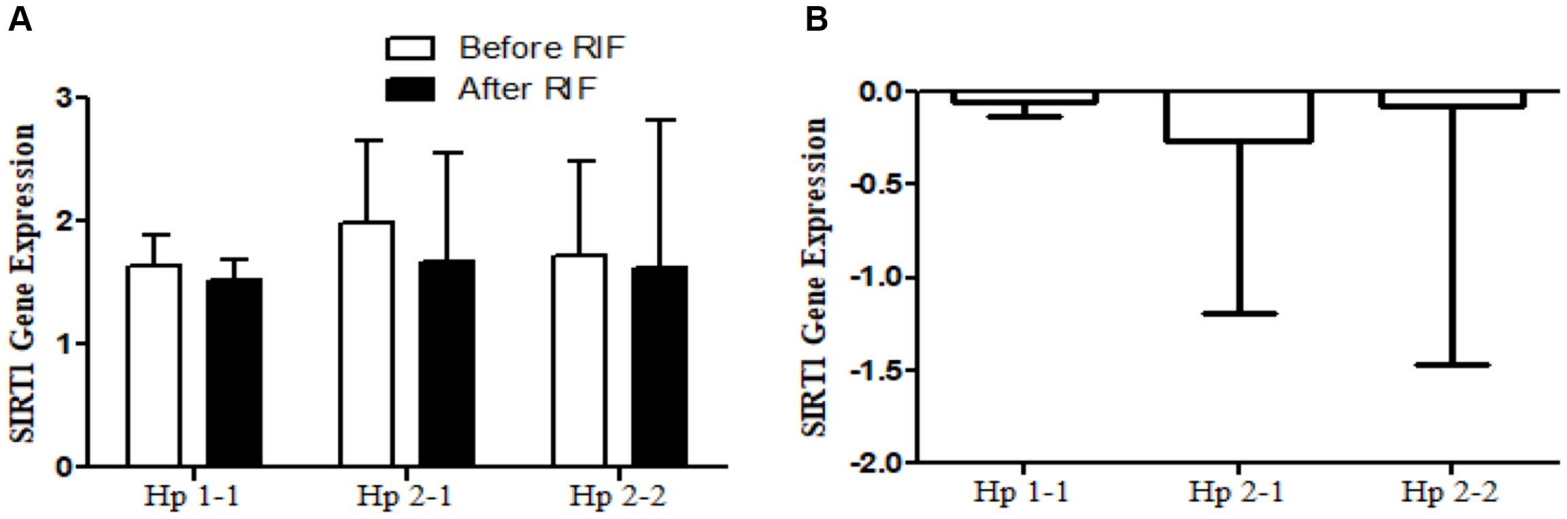
**(A)** Changes in *SIRT1* gene expressions before and after RIF based on Hp genotype. **(B)** Mean of differences in *SIRT1* gene expressions before and after RIF based on Hp genotype.

**Figure 7:**
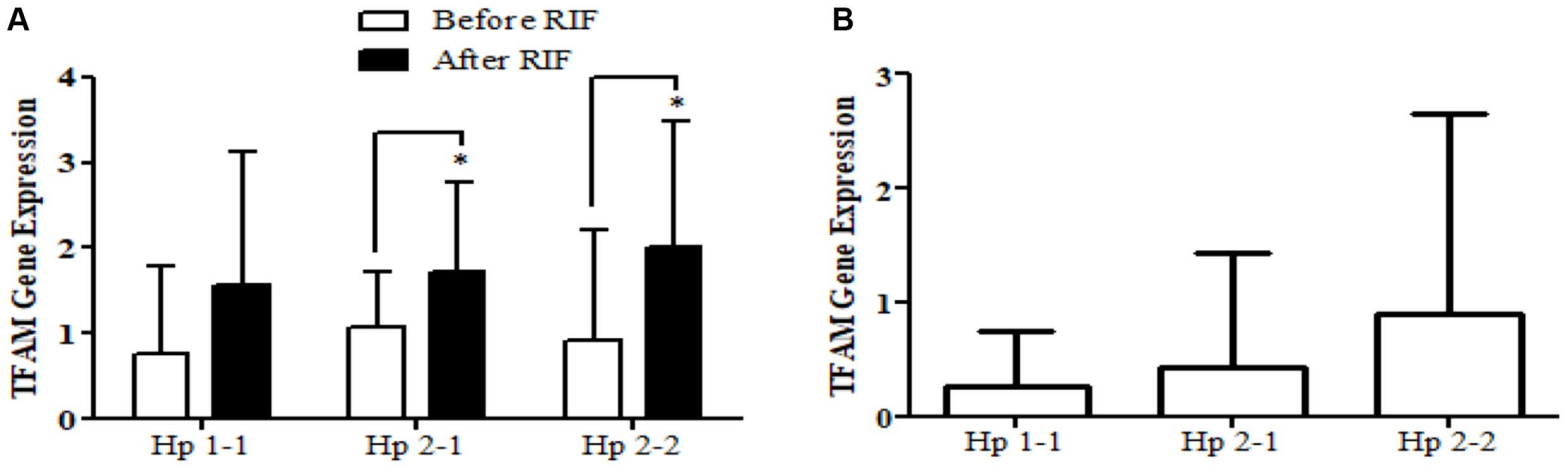
**(A)** Changes in *TFAM* gene expressions before and after RIF based on Hp genotype. **(B)** Mean of differences in *TFAM* gene expressions before and after RIF based on Hp genotype.

Similarly, the *SOD2* gene expression revealed a significant increase in participants with the Hp2-2 polymorphism genotype, not the other two genotypes (Figure 5). Further, the means of differences for *SOD2* gene expressions for the three polymorphism genotypes Hp showed a trend of increase in Hp1-1, Hp2-1, and Hp2-2 but without significant changes. Regarding *TFAM* gene expressions, Figure 6 depicts significant increases for Hp2-1 and Hp2-2 genotypes at the end of RIF in comparison with the levels of expression before. Alongside, the mean of difference for the expression of the *TFAM* gene showed a clear trend of increment associated with the values of the expression through Hp1-1, Hp2-1, and Hp2-2, respectively, but without significant changes (Figure 7).

**Figure 8:**
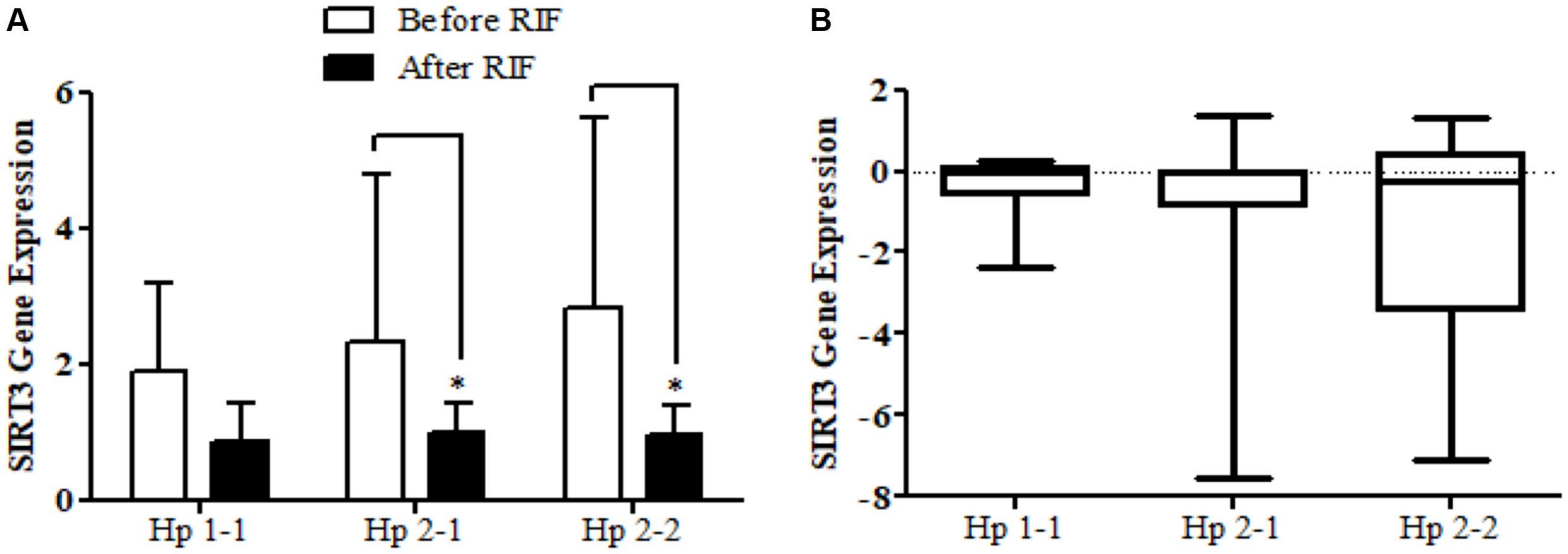
**(A)** Changes in *SIRT3* gene expressions before and after RIF based on Hp genotype. **(B)** Mean of differences in *SIRT3* gene expressions before and after RIF based on Hp genotype.

The results show no significance for *SIRT1* gene expression values in Hp1-1, Hp2-1, and Hp2-2 genotypes as individual groups after RIF in comparison to values before RIF (Figure 6). Also, with no significant values for *SIRT1* gene expression in the mean of the difference between Hp polymorphism genotypes. Unlike, the result expressed significantly lower values in *SIRT3* gene expression for Hp 2-1 and Hp2-2 genotypes and no significant differences in Hp1-1 individual groups after RIF in corresponding to values before RIF (Figure 8). Also, the *SIRT3* gene expression means of difference values between Hp polymorphism genotypes showed no significant differences (Figure 8).

## Discussion

This groundbreaking research investigates for the first time the changes in genetic expressions implicated in the regulation of inflammation and antioxidant defense enzyme system (*CD163*, *TFAM*, *SOD2*, and *Nrf2*), as well as metabolic and cellular regulation (*SIRT1* and *SIRT3*). Notably, our study showed that *CD163* gene expression levels were significantly upregulated after RIF, which supports our hypothesis that RIF had a positive effect in modulating pro-and anti-inflammatory markers. However, there may be other unexamined factors that have an impact on these factors during Ramadan, such as changes in circadian rhythm and physical activity, which may be markers, as reported in non-fasting research (28). Moreover, we report that key antioxidant defense system-controlling genes (*SOD2*, *TFAM*, and *Nrf2*) exhibited significantly elevated expression, highlighting their role in mitigating OS during fasting-induced physiological stress. This gene expression pattern aligns with findings from a meta-analysis showing decreased inflammatory and OS markers upon the observance of RIF (10). The significance of *SOD2* in counteracting the damaging effect of reactive oxygen species generated by mitochondria upon cellular respiration was confirmed in the present study. Moreover, no significant correlations were found between the genetic expressions of any of the five tested genes and the independent variables (sex, waist circumference, and BMI) except for *SOD2* and caloric intake. The *SOD2* expression was significant (*p < 0*.*05*) and directly associated with increased caloric intake (>2,000 kcal/day vs. <2,000 kcal per day) among fasting participants during Ramadan. The upregulation of the antioxidant defense enzyme genes (*TFAM*, *SOD2*, and *Nrf2*) augmented the anti-inflammatory status of fasting individuals.

*Nrf2* has emerged nowadays as a pivotal link between managing antioxidant gene expression during stress response and cell survival. It regulates the expression of genes encoding antioxidant enzymes, detoxification enzymes, and other cytoprotective proteins. *Nrf2* activity is influenced by various factors, including fasting and dietary components. Intermittent fasting can activate *Nrf2*, leading to increased expression of antioxidant enzymes like SOD2, which helps mitigate oxidative damage (29, 30). Therefore, it is reasonable that *SOD2* is overexpressed in parallel with the higher expression of the *Nrf2* gene following RIF, which may be explained by the direct effect of *Nrf2* on *SOD2* expression and activity.

While the overexpression of antioxidative stress genes during RIF might raise concerns about increased ROS production, Ristow and Schmeisser (31) defended this argument by reporting that several longevity-promoting modifications, such as IF and caloric restriction, may converge by causing activation of mitochondrial oxygen consumption to promote the increased formation of ROS. These dietary interventions may serve as molecular signals to exert downstream impacts that ultimately trigger endogenous defense mechanisms, which culminate in improved stress resistance and longevity, an adaptive response called mitohormesis or mitochondrial hormesis. Moreover, RIF increases *SOD2* expression, enhancing the cell’s ability to cope with OS. *SIRT1* and *SIRT3*, as part of the Sirtuins family of NAD^+^-dependent protein deacetylases, play key roles in cellular metabolism, stress response, and longevity.

We found that *SIRT1* gene expression decreased slightly after RIF, while *SIRT3* gene expression significantly decreased following RIF. The expression of Sirtuins is enhanced upon acute/chronic inflammation. Our findings may explain the lower expression of *SIRT1* and *SIRT3* upon RIF due to the modulation of inflammatory markers, as anti-inflammatory markers were improved, as reported in our previous study. In the context of this study, *SIRT3*, the primary mitochondrial deacetylase enzyme, orchestrates fatty acid breakdown during prolonged fasting. While *SIRT1* expression remained unchanged, *SIRT3* showed a significant decrease post-RIF. These results could be explained by the lack of significant differences in fat intake and total energy between baseline and post-Ramadan, alongside excessive simple sugar intake throughout feeding night hours of Ramadan month, as shown in the dietary intake of the present study (data not shown). Several studies have reported that *SIRT1* and *SIRT3* were overexpressed following excessive caloric restriction and prolonged fasting (32, 33), which is unlike in RIF where it involves 12–17 fasting hours/day without any intake (including water), with the remainder of the night hours available for eating without restriction (5).

*SIRT3* gene expression is acutely responsive to a cell’s primary nutrient availability. Caloric restriction, exercise, and fasting enhance SIRT3 expression in diverse tissues (34–36). Conversely, another study indicated reduced SIRT3 during skeletal muscle fasting (37), contrasting with reports of decreased SIRT3 in high-fat-fed rodents and humans with metabolic syndrome (38–40). Our study notes increased carbohydrate, total sugar, and total fat consumption during Ramadan night hours, potentially explaining the lowered SIRT3 gene expression during Ramadan fasting.

To our knowledge, this is the first study to investigate the role of the Hp genotype (Hp1-1, Hp2-1, and Hp2-2) in *CD163* gene expression, antioxidants enzymes including (*Nrf2*, *SOD2*, and *TFAM*), and cellular regulatory genes (*SIRT1* and *SIRT3*) in response to RIF. Our results showed a significant enhancement of CD163 gene expression among Hp2-2 individuals compared with Hp2-1 and Hp1-1 individuals. These results are confirmed by using the mean of difference statistical methods, which shows a significant response of Hp2-2 to RIF compared to Hp1-1 and Hp2-1. This may explain the marked improvement in action and response to RIF among individuals with obesity, where Hp2-2 individuals had a stronger reaction and secreted more anti-inflammatory markers in our previous study (22). Additionally, we previously showed that IL-10 has significantly increased after RIF. This could be correlated with another study, which reported that CD163 expression is controlled and upregulated considerably by IL-10. They also noted that CD163 has an anti-inflammatory effect by inhibiting phorbol ester-induced human T-lymphocyte activation, resulting in the weakening of the immune response to the inflammatory mediator (41). Another explanation for how high levels of CD163 modulate the anti-inflammatory effect is by directly inducing intracellular signaling that leads to the secretion of anti-inflammatory cytokines. Second and perhaps even more important, the CD163-mediated delivery of hemoglobin to the macrophage may trigger an anti-inflammatory response because heme metabolites have potent anti-inflammatory effects (42). These findings demonstrated the benefits of RIF in modulating and improving the systemic inflammation of individuals with obesity, especially those with the Hp2-2 genotype.

Similarly, the antioxidant enzyme *Nrf2* gene expression demonstrated a significant increment in individuals with obesity in the Hp1-1, Hp2-1, and Hp2-2 groups after RIF compared with before RIF; the values were more than five-fold in Hp1-1 and Hp2-1 and more than eight-fold in Hp2-2. In addition, the mean difference for *Nrf2* gene expression between Hp genotypes revealed an insignificant trend of increment associated with Hp1-1, Hp2-1, and Hp2-2. Similarly, the antioxidant enzyme *SOD2* gene expression revealed significant enhancement in the Hp2-1 and Hp2-2 groups following RIF. For *Nrf2*, the mean differences in *SOD2* and *TFAM* between Hp genotype groups showed an important trend of increment in Hp1-1, Hp2-1, and Hp2-2, in that order. These results support the hypothesis of a previous study that suggested that the genotype of Hp has a crucial role in modulating the oxidative-antioxidative status in both obesity and diabetes (43) and reveal the positive impact/response of RIF based on the inflammatory status, which significantly elevated in the higher proinflammatory genotype Hp2-2 in order to modulate the inflammatory response. In another large-scale research, where they studied the levels of many antioxidant enzymes, including SOD, in 165 diabetic patients, they reported that diabetic patients with Hp2-2 genotype have lower levels of SOD compared to Hp2-1 and Hp1-1 (25). We suggest that fasting could reverse this and rather lead to increased levels of antioxidants like SOD enzyme in diabetic patients. In contrast, we observed significantly lower values in *SIRT3* gene expression for the Hp2-1 and Hp2-2 genotypes and no significant differences for Hp1-1 after RIF compared with before RIF. No significant difference was observed for *SIRT1* in any Hp genotype in response to RIF.

We can claim that this is the first study that examined the changes in the gene expressions of *Nrf2*, *SOD2*, *TFAM*, *SIRT1*, and *SIRT3* in the context of IF research and based on the Hp genotype. This uniqueness arises from the fact that RIF is the only dry and diurnal fasting model among the different IF models. While IF is known to trigger various cellular pathways that can impact health and longevity, these mechanisms are regulated partly by key players at the molecular and cellular pathways, which are *Nrf2*, *TFAM*, *SOD2*, *SIRT1*, and *SIRT3*. These bioactive molecules, in summary, are key players in cellular responses to IF. Their interplay helps regulate OS, mitochondrial function, and cellular metabolism, contributing to the health-promoting effects of IF.

A few limitations should be considered when interpreting the findings of the current study. First, causality cannot be inferred due to the observational prospective design and the lack of a control group. Undetected confounding factors could contribute to the downregulation or upregulation of the tested genes during RIF. Further, changes in circadian rhythm and sleep patterns, known to affect gene expression, are among the potential factors influencing these changes (44). Although physical exercise levels did not significantly change during Ramadan compared to the pre-fasting stage, physical activity could still have a substantial impact, and future studies should incorporate objective measurements of exercise levels. Further, seasonal variations should be considered in future research, as the month of Ramadan spans different solar seasons, which are known to influence gene expressions (45). Lastly, the findings from the current work cannot be generalized to other ethnicities or the general population. Ethnic variations should be considered and examined in the context of the effects of dawn-to-dusk intermittent fasting on various health outcomes.

## Conclusion

Our findings suggest that the observance of 4 weeks of dawn-to-dusk IF during Ramadan could significantly alter the expression of genes associated with inflammation and OS regulation in individuals with obesity. Specifically, the Hp2-1 and Hp2-2 genotypes of the Hp polymorphism demonstrated a more pronounced antioxidative and anti-inflammatory response to the RIF model compared to the Hp1-1 genotype. Consequently, a four-week period of dawn-to-dusk IF during Ramadan may enhance health outcomes in obese individuals by modulating and improving their oxidative and inflammatory mechanisms. These results underscore the potential health benefits of RIF and its role in managing obesity-related inflammation and oxidative stress.

## Data Availability

The raw data supporting the conclusions of this article will be made available by the authors, without undue reservation.
